# Prediction of the Rehabilitation Duration and Risk Management for Mild-Moderate COVID-19

**DOI:** 10.1017/dmp.2020.214

**Published:** 2020-06-24

**Authors:** Qiong-Na Zheng, Mei-Yan Xu, Yong-Le Zheng, Xiu-Ying Wang, Hui Zhao

**Affiliations:** 1 Department of Infectious Diseases, Affiliated Yueqing Hospital, Wenzhou Medical University, Yueqing, China

**Keywords:** mild-moderate COVID-19, predictive model, refined risk management, rehabilitation duration, tailored clinical decisions-making

## Abstract

**Objectives:**

More than 80% of coronavirus disease 2019 (COVID-19) cases are mild or moderate. In this study, a risk model was developed for predicting rehabilitation duration (the time from hospital admission to discharge) of the mild-moderate COVID-19 cases and was used to conduct refined risk management for different risk populations.

**Methods:**

A total of 90 consecutive patients with mild-moderate COVID-19 were enrolled. Large-scale datasets were extracted from clinical practices. Through the multivariable linear regression analysis, the model was based on significant risk factors and was developed for predicting the rehabilitation duration of mild-moderate cases of COVID-19. To assess the local epidemic situation, risk management was conducted by weighing the risk of populations at different risk.

**Results:**

Ten risk factors from 44 high-dimensional clinical datasets were significantly correlated to rehabilitation duration (*P* < 0.05). Among these factors, 5 risk predictors were incorporated into a risk model. Individual rehabilitation durations were effectively calculated. Weighing the local epidemic situation, threshold probability was classified for low risk, intermediate risk, and high risk. Using this classification, risk management was based on a treatment flowchart tailored for clinical decision-making.

**Conclusions:**

The proposed novel model is a useful tool for individualized risk management of mild-moderate COVID-19 cases, and it may readily facilitate dynamic clinical decision-making for different risk populations.

Since the outbreak in late December 2019, the current epidemic of coronavirus disease 2019 (COVID-19) caused by severe acute respiratory syndrome coronavirus-2 (SARS-CoV-2)^1^ remains severe worldwide.^2^ The number of confirmed cases and deaths has been increasing markedly,^3^ and mild and moderate cases have accounted for more than 80% of all cases.^4,5^ This indicates that the key to containing the outbreak and preventing further spread of the disease is to increase the effectiveness of risk management for mild-moderate COVID-19 cases. Compared with severe cases, the most valuable strategy in the management of mild-moderate COVID-19 may be the establishment of Fangcang shelter hospitals schema rather than intensive care units, isolating mild-moderate patients while providing health care, surveillance, food, shelter, and social activities.^6^ If the rehabilitation duration of mild-moderate COVID-19, from hospital admission to discharge, can be effectively predicted, it will facilitate refined risk management on the basis of personalized medicine according to local epidemic situations, which is helpful in assessing and controlling medical resources consumption and costs.

The current risk evaluation of mild-moderate COVID-19 mainly focuses on case reports of clinical features, analyses of single clinical factors of COVID-19, and multiple factor modeling. Case reports have covered numerous mild-moderate COVID-19 typical and atypical features, including clinical symptoms, laboratory tests, imaging findings, and immunological features.^7-9^ Analyses of risk factors have revealed relationships between single factors and clinical outcomes.^10-12^ Prediction models that combine multiple variables or features to estimate the risk of individuals being infected or having a poor outcome could assist medical staff in triaging patients when allocating limited health-care resources.^13-15^ These studies have well reflected certain features of mild-moderate COVID-19, but there is still no available method to effectively predict the rehabilitation duration of mild-moderate COVID-19. Thus, the challenge of refined risk management for mild-moderate COVID-19 remains.

This study aimed to develop a predictive model for the rehabilitation duration of mild-moderate COVID-19 based on analyses of clinical risk factors, to increase the level of expertise in facilitating refined risk management for the precision delivery of health-care services and tailored decision-making.

## METHODS

### Patients

**FIGURE 1:**
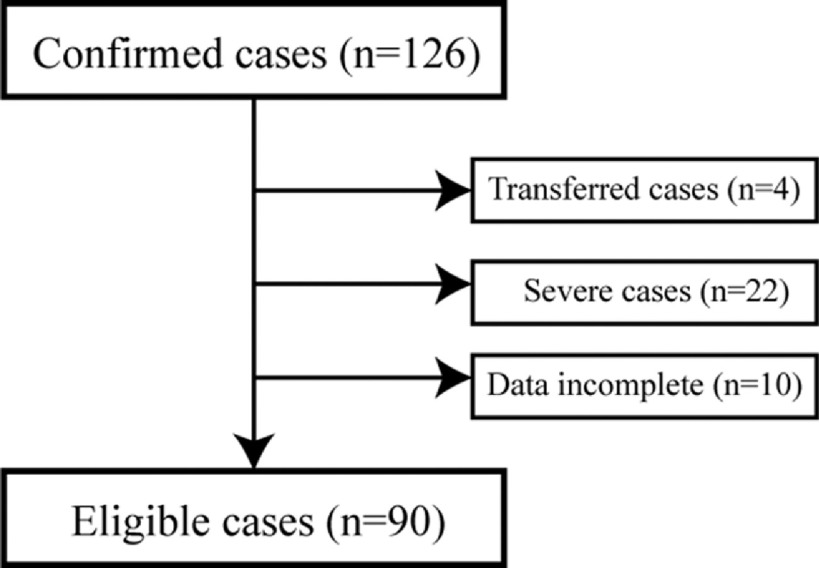
Flowchart of Inclusion and Exclusion

Consecutive inpatient patients with COVID-19 in the Affiliated Yueqing Hospital, Wenzhou Medical University, from January to February 2020 were retrospectively enrolled. Patients were excluded from this study if they (1) had transferred to other hospitals before cure, (2) were classified as severe cases according to the guidelines released by the National Health Commission of China,^16^ and (3) had incomplete data. The inclusion–exclusion process is summarized in Figure 1.

Confirmed cases with COVID-19 were from consecutive inpatients in the Affiliated Yueqing Hospital, Wenzhou Medical University from January to February 2020.

Cases were classified according to the Diagnosis and Treatment Protocol for Novel Coronavirus Pneumonia (Trial Version 7), National Health Commission of China^16^: (1) mild cases, mild clinical symptoms, and no sign of pneumonia on imaging; (2) moderate cases, showing fever, and respiratory symptoms with radiological findings of pneumonia; and (3) severe cases, that is, (a) respiratory distress (≥ 30 breaths/min), (b) oxygen saturation ≤ 93% at rest, (c) arterial partial pressure of oxygen (PaO2)/fraction of inspired oxygen (FiO2) ≤ 300 mmHg (l mmHg = 0.133 kPa), and (d) cases with chest imaging that shows obvious lesion progression > 50% within 24-48 h.

The rehabilitation duration is the time from hospital admission to discharge. Discharge criteria according to the Diagnosis and Treatment Protocol for Novel Coronavirus Pneumonia (Trial Version 7), National Health Commission of China^16^: (1) body temperature is back to normal for more than 3 d; (2) respiratory symptoms improve noticeably; (3) pulmonary imaging shows obvious improvement of inflammation; (4) nucleic acid tests negative twice consecutively on respiratory tract samples, such as sputum and nasopharyngeal swabs (sampling interval being at least 24 h).

Collectible large-scale datasets were extracted from the immediate blood-test results upon admission, including demographics, etiology, clinical conditions, blood gas analysis, blood cell analyses, blood biochemistry (hepatic function, renal function, electrolytes, lipid, and glucose), and coagulation function.

### Statistical Analysis

Statistical analyses were performed using SPSS 25.0 (IBM, USA). Continuous variables were expressed as mean and SD (mean ± SD), and categorical variables were expressed as frequencies and/or percentages. The correlations between each clinical factor and the rehabilitation duration were analyzed by bivariate correlations. *P* < 0.05 was considered significant. Significant clinical factors were considered as risk factors for further modeling. Through the multivariable stepwise linear regression analysis, a model was developed for predicting the individualized rehabilitation duration of each mild-moderate case. According to risk prediction with this model, risk management was based on local epidemic situations for tailored decision-making.

## RESULTS

### Demographic and Clinical Characteristics of Mild-Moderate COVID-19 Cases

A total of 90 eligible patients from 126 consecutive patients were enrolled in this study (Figure 1). The rehabilitation duration was 17.2 ± 5.2 d. There were 10 significant risk factors (*P* < 0.05; shown in Table 1) from 44 high-dimensional clinical datasets (Supplemental Table S1, which is available online).

**TABLE 1:** Characteristics of Cases

	Mean ± SD (n = 90)	R	P-value
Rehabilitation duration (days)	17.2 ± 5.2	–	–
Age (years)	49.4 ± 12.5	− 0.075	0.482
Sex [male, n (%)/female, n (%)]	46 (51.1) / 44 (48.9)	− 0.199	0.060
PaCO_2_ (mmHg)	37.8 ± 3.8	0.287	0.006
Lactic acid (mmol/L)	1.7 ± 0.6	0.225	0.033
TBIL (μmol/L)	10.6 ± 5.9	0.224	0.034
AST (U/L)	26.4 ± 13.2	− 0.211	0.046
BUN (mmol/L)	3.7 ± 1.1	0.227	0.031
Triglyceride (mmol/L)	1.7 ± 1.2	0.234	0.027
K (mmol/L)	4.3 ± 0.5	0.281	0.007
WBC (10^9/L)	4.8 ± 1.7	0.317	0.002
HGB (g/L)	136.4 ± 15.0	0.216	0.041
NEUT (10^9/L)	2.8 ± 1.4	0.230	0.029
CD4+ (/ul)	639.6 ± 398.7	0.112	0.292
CD8+ (/ul)	393.8 ± 177.9	0.129	0.227

Abbreviations: PaCO2: partial pressure of carbon dioxide in artery; TBIL: total serum bilirubin; AST: aspartate aminotransaminase; BUN: blood urea nitrogen; K: serum potassium; WBC: white blood cell; HGB: hemoglobin; NEUT: neutrophils.

### Predictor Selection and Model Development

Through multivariable stepwise linear regression, 5 risk predictors (white blood cell [WBC], partial pressure of carbon dioxide [PaCO2], serum potassium [K], total bilirubin [TBIL], and aspartate aminotransaminase [AST]) from 10 risk factors were incorporated to develop a predictive model for the rehabilitation duration of mild-moderate COVID-19 cases (F = 11.055; *P* < 0.001, and adjusted R^2^ = 0.361). The detail of risk predictor parameters with respect to the model is shown in Table 2.

**TABLE 2:** Multiple Linear Regression Analysis of Predictor Parameters With Respect to Rehabilitation Duration

	df	SS	MS	F	*P* value
Regression	5	968.156	193.631	11.055	< 0.001
Residual	84	1471.335	17.516		
Total	89	2439.491			
**Multiple Linear Regression**					
	**Coefficient**	**SE**	**Beta**	**95% CI**	**P value**
Constant	−21.696	7.198		−36.010 – −7.382	0.003
WBC	0.908	0.277	0.297	0.358 – 1.458	0.001
PaCO2	0.428	0.120	0.312	0.189 – 0.667	0.001
K	4.209	1.014	0.372	2.192 – 6.226	< 0.001
TBIL	0.251	0.082	0.282	0.087 – 0.415	0.005
AST	−0.086	0.036	− 0.217	− 0.157 – −0.015	0.018

**Note:** R^2^ = 0.397; adjusted R^2^ = 0.361.

Abbreviations: df: degrees of freedom; SS: sum of squares; MS: mean squares; CI: confidence interval; WBC: white blood cell; PaCO2: partial pressure of carbon dioxide in artery; K: serum potassium; TBIL: total serum bilirubin; AST: aspartate aminotransaminase.

### Presentation of Refined Risk Management

**FIGURE 2:**
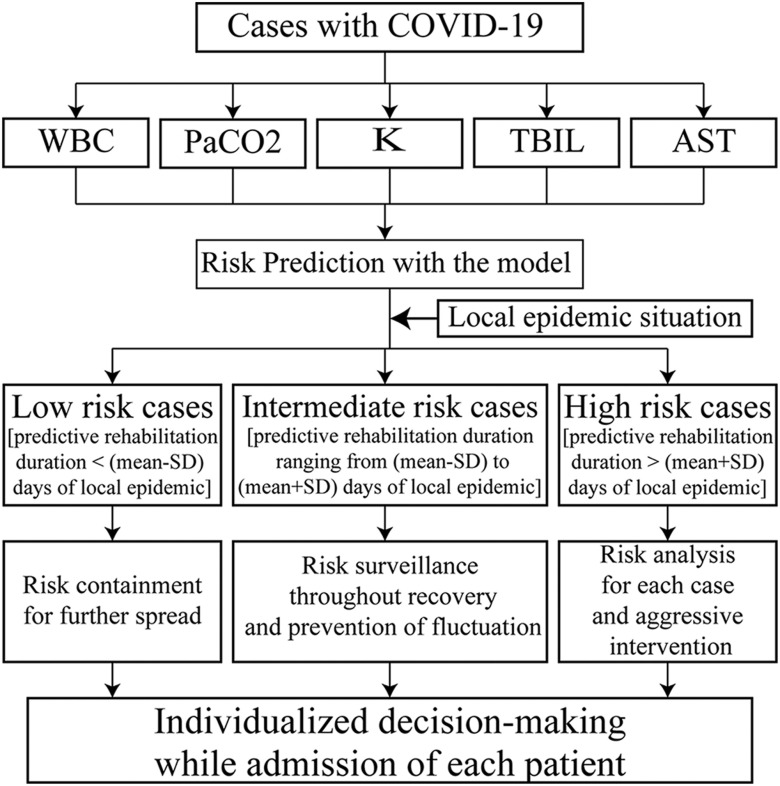
Treatment Flow-Chart of Risk Management for Mild-Moderate COVID-19

Using the predictive model, the rehabilitation duration of each mild-moderate case can be individually calculated. Therefore, the treatment flow-chart of refined risk management for mild-moderate COVID-19 was proposed. Weighing the mean and SD of the rehabilitation duration of local overall cases in different regions, mild-moderate cases of each region can be classified for 3 independent risk populations based on the time-consumption threshold: low risk of < (mean - SD) days of the local overall rehabilitation duration, intermediate risk of (mean ± SD) days of the local overall rehabilitation duration, and high risk of > (mean + SD) days of the local overall rehabilitation duration. According to this risk stratification, mild-moderate cases can be further subdivided into different risk populations and tailored clinical decision-making can be conducted. If the predictive rehabilitation duration of 1 mild-moderate case of COVID-19 varies during the recovery process, tailored decision-making can be adjusted dynamically (Figure 2). Based on the predictive system, it may readily facilitate more effective risk management of mild-moderate COVID-19.

Based on the mean and SD of the rehabilitation duration of local overall cases in different regions, the threshold time-consumption of each region can be classified for 3-independent risk populations: low risk of < (mean - SD) days of the local overall rehabilitation duration, intermediate risk of (mean ± SD) days of the local overall rehabilitation duration, and high risk of > (mean + SD) days of the local overall rehabilitation duration. WBC, white blood cell; PaCO2, partial pressure of carbon dioxide in artery; K, serum potassium; TBIL, total serum bilirubin; AST, aspartate aminotransaminase.

## DISCUSSION

In this study, we analyzed significant clinical risk factors and developed a model based on predicting rehabilitation duration for the refined risk management of mild-moderate COVID-19. To our knowledge, this study is the first to predict the rehabilitation duration for mild-moderate COVID-19 based on large-scale datasets of clinical factors. The rehabilitation duration can be easily calculated using the risk model for 1 mild-moderate case of COVID-19 (F = 11.055, *P* < 0.001, and adjusted R^2^ = 0.361). According to the risk stratification of the local epidemic, tailored clinical decisions-making can be conducted, and strategic risk management can be derived. The model incorporated 5 clinical indices of WBC, PaCO^2^, K, TBIL, and AST, from blood gas analysis, blood cell analyses, hepatic function, and electrolytes, respectively. These indices reflect information on various aspects of mild-moderate COVID-19 during the recovery course. Therefore, these 5 predictors are independent factors that are related to the rehabilitation risk of mild-moderate COVID-19, and can be used as simple and effective prognostic evaluation indicators.

Among the 5 predictors incorporated into the model, WBC counts are often considered as a marker of systemic inflammation and immune system health.^17^ The acute variation of WBC counts is related to infections or other environmental exposures.^18^ A study found^19^ that the WBC count increased in the group of COVID-19 patients who died during hospitalization; comorbid bacterial or fungal infection might have occurred in these patients, causing the WBC count to increase. Our study also validated that WBC count can be used as a predictor of rehabilitation duration. Interestingly, CD4+ and CD8+ T cells play protective roles in coronavirus infection^13,20,21^ but were not incorporated into our model. Previous studies found that, during the acute phase of SARS-CoV infection in humans, CD4+ and CD8+ T cell counts were decreased.^22-24^ SARS-CoV-2 also presented similar characteristics, because viral infection causes persistent consumption and/or insufficient regeneration of lymphocytes.^19^ Therefore, CD4+ and CD8+ T cell counts vary in response to SARS-CoV-2 infection in the acute phase and may be far from satisfactory for protection during the entire recovery period.

Compared with PaO2/FiO2, which is widely accepted for quantifying oxygenation in the evaluation and monitoring of severe cases of COVID-19,^25^ studies regarding PaCO2 on COVID-19 are scarce. PaCO2 reflects the balance between the production and elimination of carbon dioxide (CO2) and is related to alveolar ventilation. Increased PaCO2 levels indicate the inefficiency of the lungs to eliminate CO2 and increase in pulmonary dead space.^26^ Therefore, several scholars have proposed to use intermediate tidal volume instead of low tidal volume to correct the inadequacy of ventilation in COVID-19 cases.^27^ On the other hand, a study from Xixi Hospital in Hangzhou reported^28^ that patients with COVID-19 had low K level, especially 1 case of extended hospitalization, but these levels increased persistently after treatment. In our study, PaCO2 and K were significantly related to the rehabilitation duration, and the PaCO2 level of all 90 cases and K levels in most cases were normal. Related research regarding 2 predictors and COVID-19 is rare. Our study suggests that the value of PaCO2 and K on COVID-19 should be further explored, as they may become important predictors of clinical outcome, even if at the normal level, and may be suitable predictors for all COVID-19 cases rather than only severe cases.

At least 7 relatively large-scale case studies have reported^29-35^ that 2-11% of COVID-19 cases had liver comorbidities, and 14-53% of cases have reported abnormal liver function during disease progression. Severe COVID-19 cases have higher rates of liver dysfunction.^36^ Liu et al. determined^37^ that cytokine storm syndrome was likely to emerge in severe cases and could result in abnormal serum transaminase and bilirubin levels. AST and TBIL were incorporated into our model, which may reflect the levels of cytokine release in mild-moderate COVID-19 cases. Moreover, another study^38^ found that there was no correlation between increases in transaminase and TBIL (R = −0.006; *P* = 0.972). In our study, it is not difficult to understand that TBIL was positively correlated with rehabilitation duration; but of interest, all transaminases (including AST) were negatively correlated with rehabilitation duration (shown in Supplemental Table S1). Transaminases mainly exist in hepatocytes and are released when hepatocellular damage occurs, but the excessively low level of transaminases is not a favorable signal in disease progression, such as in liver failure.^39^ Because transaminases are indispensable catalysts for the normal operation of the liver, the moderate transaminase levels in mild-moderate cases reflects the activity of hepatocytes (not limited to COVID-19).

In this study, with the help of statistical analysis and comprehensive analysis of 5 conventional laboratory indicators, a mathematical model was developed to effectively predict the rehabilitation duration of mild-moderate COVID-19 cases without complicated and expensive detection techniques. As these tests can be performed in grass-root medical hospitals, and the results can be derived quickly, the risk model has wide clinical applicability. If the local rehabilitation duration is 14 ± 5 d (means ± SD), when the predicted rehabilitation duration of 3 mild-moderate cases with COVID-19 is 7, 10, and 20 d, the 3 cases have low, intermediate, and high risks, respectively. Accordingly, the strategy of individualized treatments should focus on containing the further spread in low-risk cases, and surveilling the complete recovery process and preventing the disease fluctuation in intermediate risk cases, while it is necessary to analyze the reasons for each high-risk case and perform aggressive interventions. If the predictive rehabilitation duration of 1 mild-moderate COVID-19 case varies during the recovery process, tailored decisions-making can be adjusted dynamically (Figure 2). Based on this refined risk management system, frontline medical staff can increase their levels of expertise regarding mild-moderate COVID-19, which fits the current trend toward personalized medicine. More importantly, this may help to assess the cost of local epidemic control in the context of big data.

There are several limitations in this study. First, limited by the outbreak scale of our local epidemic, the sample size in this study was not large enough. Second, the R^2^ and adjusted R^2^ of the model were not significant, and we cannot avoid potential biases as a retrospective study. Third, because all cases in this study were from our local region, it is unclear whether the characteristics were consistent with those of other regions. Finally, as a retrospective study, we did not obtain the re-examined test of 5 risk predictors at the same timepoint to validate the model from other different timepoints to discharge. This is the first study of its kind, there is no similar model for reference, and the proposed model may be further optimized after incorporating more valuable variables and larger cases. Therefore, in the next step, we will strive to conduct a multi-center and multi-area research for enrolling a larger number of cases to validate and improve our model for the rehabilitation duration prediction from any timepoint to discharge and early warning if the disease varies.

## CONCLUSIONS

By collecting large-scale datasets from clinical practices, this study systematically analyzed the correlation between clinical risk factors and rehabilitation duration of mild-moderate COVID-19 cases and further developed a risk model for predicting the rehabilitation duration of mild-moderate COVID-19 cases. The proposed model is a useful tool of refined risk management for facilitating timely tailored clinical decision-making for mild-moderate COVID-19 and may help assess the cost of local epidemic control in the context of big data.

## Availability of Data and Materials

The datasets used and/or analyzed during the current study are available from the corresponding author on reasonable request.

## Supplementary material

For supplementary material accompanying this paper visit http://dx.doi.org/10.1017/dmp.2020.214. click here to view supplementary material
