# Supplementary material for: Prediction of the Rehabilitation Duration and Risk Management for Mild-Moderate COVID-19
Source: Disaster Med Public Health Prep. 2020 Jun 24:1–6. doi: 10.1017/dmp.2020.214 (PMC7369334; doi:10.1017/dmp.2020.214)
Supplement: Supplementary file 1 [file S1935789320002141sup001.docx]

**Table S1 Characteristics of mild-moderate cases with COVID-19**

|  | **Mean ± SD (n = 90)** | **R** | **P** |
| --- | --- | --- | --- |
| Convalescence (days) | 17.2 ± 5.2 | - | - |
| Age (years) | 49.4 ± 12.5 | - 0.075 | 0.482 |
| Sex ［male, n (%) / female, n (%)］ | 46 (51.1) / 44 (48.9) | - 0.199 | 0.060 |
| Fever ［n (%)］ | 27 (30) | 0.003 | 0.975 |
| PH value | 7.4 ± 0.3 | – 0.151 | 0.157 |
| PaO_2_ (mmHg) | 101.5 ± 18.3 | – 0.132 | 0.214 |
| Oxygenation index (mmHg) | 428.0 ± 49.9 | – 0.200 | 0.059 |
| PaCO_2_ (mmHg) | 37.8 ± 3.8 | 0.287 | 0.006 |
| Lactic acid (mmol/l) | 1.7 ± 0.6 | 0.225 | 0.033 |
| D-dim (mg/L) | 0.7 ± 1.5 | - 0.158 | 0.136 |
| TBIL (μmol/L) | 10.6 ± 5.9 | 0.224 | 0.034 |
| DBIL (μmol/L) | 4.4 ± 2.5 | 0.168 | 0.113 |
| ALB (g/L) | 41.2 ± 3.7 | 0.041 | 0.698 |
| ALT (U/L) | 27.7 ± 25.3 | - 0.090 | 0.399 |
| AST (U/L) | 26.4 ± 13.2 | - 0.211 | 0.046 |
| GGT (U/L) | 39.8 ± 42.2 | - 0.084 | 0.431 |
| AKP (U/L) | 62.8 ± 17.2 | 0.014 | 0.896 |
| LDH (U/L) | 223.7 ± 58.9 | - 0.150 | 0.158 |
| ADA (U/L) | 14.5 ± 6.3 | - 0.047 | 0.659 |
| FBG (mmol/L) | 5.9 ± 2.0 | -0.035 | 0.742 |
| Cr (μmol/L) | 65.1 ± 17.9 | 0.195 | 0.066 |
| BUN (mmol/L) | 3.7 ± 1.1 | 0.227 | 0.031 |
| Cholesterol (mmol/L) | 4.4 ± 0.9 | 0.004 | 0.970 |
| Triglyceride (mmol/L) | 1.7 ± 1.2 | 0.234 | 0.027 |
| K (mmol/L) | 4.3 ± 0.5 | 0.281 | 0.007 |
| Na (mmol/L) | 138.0 ± 14.4 | 0.118 | 0.266 |
| Cl (mmol/L) | 100.2 ± 3.1 | - 0.154 | 0.148 |
| Ca (mmol/L) | 2.2 ± 0.1 | 0.128 | 0.230 |
| CK (U/L) | 95.8 ± 84.1 | - 0.026 | 0.811 |
| CK-MB (U/L) | 18.1 ± 6.3 | 0.028 | 0.791 |
| WBC (10^9/L) | 4.8 ± 1.7 | 0.317 | 0.002 |
| RBC (10^12/L) | 4.5 ± 0.51 | 0.195 | 0.065 |
| HGB (g/L) | 136.4 ± 15.0 | 0.216 | 0.041 |
| PLT (10^9/L) | 203.7 ± 65.8 | 0.122 | 0.253 |
| NEUT (10^9/L) | 2.8 ± 1.4 | 0.230 | 0.029 |
| LYM (10^9/L) | 1.5 ± 0.6 | 0.186 | 0.079 |
| Mon (10^9/L) | 0.7 ± 1.3 | 0.014 | 0.893 |
| Eos (10^9/L) | 0.1 ± 0.1 | 0.185 | 0.080 |
| NEUT % | 56.6 ± 10.3 | 0.004 | 0.967 |
| LYM % | 31.2 ± 8.6 | - 0.086 | 0.423 |
| Mon % | 10.9 ± 3.1 | 0.187 | 0.078 |
| Eos % | 1.2 ± 1.7 | 0.052 | 0.626 |
| CRP (mg/L) | 22.0 ± 12.6 | 0.087 | 0.414 |
| CD3+ (/ul) | 1062.4 ± 479.2 | 0.132 | 0.214 |
| CD4+ (/ul) | 639.6± 398.7 | 0.112 | 0.292 |
| CD8+ (/ul) | 393.8± 177.9 | 0.129 | 0.227 |

**Abbreviations:** SD: standard deviation; PaO2: partial pressure of oxygen in artery; PaCO2: partial pressure of carbon dioxide in artery; TBIL: total serum bilirubin; DBIL: direct serum bilirubin; ALB: albumin; ALT: alanine aminotransaminase; AST: aspartate aminotransaminase; GGT: gamma-glutamyl transferase; AKP: alkaline phosphatase; LDH: lactate dehydrogenase; ADA: adenosine deaminase; FBG: fasting plasma glucose; Cr: creatinine; BUN: blood urea nitrogen; K: serum potassium; Na: serum sodium; K: serum chlorine; Ca: serum calcium; CK: creatine kinase; CK-MB: creatine kinase isoenzyme; WBC: white blood cell; RBC: red blood cell; HGB: hemoglobin; PLT: platelet; NEUT: neutrophils; LYM: lymphocytes; Mon: monocytes; Eos: eosinophils ; CRP: C-reactive protein.
